# Retrospective Cohort Study on the Impact of Travel Distance on Late-Stage Oral Cancer Treatment and Outcomes: An NCDB Analysis

**DOI:** 10.3390/cancers16152750

**Published:** 2024-08-02

**Authors:** Courtney S. Harris, Adrienne Groman, S. Lynn Sigurdson, William J. Magner, Anurag K. Singh, Vishal Gupta

**Affiliations:** 1 Roswell Park Summer Research Experience Program in Cancer Science, Roswell Park Comprehensive Cancer Center, Buffalo, NY 14263, USA; csh98@cornell.edu; 2 Roswell Park Comprehensive Cancer Center, Buffalo, NY 14263, USA; adrienne.groman@roswellpark.org (A.G.); lynn.sigurdson@roswellpark.org (S.L.S.); william.magner@roswellpark.org (W.J.M.); anurag.singh@roswellpark.org (A.K.S.); 3 College of Arts and Sciences, Cornell University, Ithaca, NY 14850, USA; 4 Weill Medical College, Cornell University, New York, NY 10065, USA

**Keywords:** oral cancer, survival analysis, retrospective studies, NCDB, travel, hospitals, numerical data

## Abstract

**Simple Summary:**

The National Comprehensive Cancer Network guidelines provide evidence-based consensus for optimal individual site- and stage-specific treatments. We evaluated the National Cancer Database (NCDB) with a cohort of 11,121 late-stage oral cancer patients. We assessed differences in treatment choices, type of facility, and survival outcome in relation to distance traveled to receive treatment. White patients were most likely to travel farthest for treatment compared to Black patients. Urban area patients traveled shorter distances than those from rural areas. Farther travel distance was associated with surgery as the primary modality and treatment at academic/research centers was associated with improved overall survival. Recognition that these factors contribute to positive outcomes may help improve survival for this patient population.

**Abstract:**

The National Comprehensive Cancer Network guidelines provide evidence-based consensus for optimal individual site- and stage-specific treatments. This is a cohort study of 11,121 late-stage oral cancer patients in the National Cancer Database from 2010 to 2016. We hypothesized that patient travel distance may affect treatment choices and impact outcome. We split travel distance (miles) into quartiles (D1–4) and assessed treatment choices, type of facility, and survival outcome in relation to distance traveled. Univariate and multivariate analyses addressed contributions of specific variables. White patients were most likely to travel farthest (D4) for treatment compared to Black patients (D1). Urban area patients traveled shorter distances than those from rural areas. Greater travel distance was associated with patients undergoing surgical-based therapies and treatment at academic centers. Patients in D1 had the lowest median survival of all distance quartiles. Surgery-based multimodality treatment (surgery and radiation) had a median survival significantly greater than for non-surgical therapy. Several factors including travel distance and treatment facility were associated with survival outcomes for late-stage oral cavity cancers. Consideration of these factors may help improve the outcome for this patient population.

## 1. Introduction

Cancer is the second-leading cause of death in the United States overall and the leading cause among people younger than 85 years. In 2024, 2,001,140 new cancer cases and 611,720 cancer deaths are projected to occur in the United States and, of those, oral cavity cancers are estimated at 36,620 cases with 7930 deaths (1.8%) [1]. They are best treated when detected early [2], but factors including late screening, tumor location, insurance status, health literacy or continued smoking may lead to some patients presenting with late-stage oral cancers [3,4,5]. Other demographic characteristics such as income and education level may also impact access to care or level of personal and professional dental care [6,7,8]. The distance patients need to travel for treatment has been discussed in relation to its impact on overall survival [9,10,11] without a clear consensus. Various aspects of travel (time, access, distance, cost) contribute to racial, ethnic, and socioeconomic disparities in access to care [12].

Although the National Comprehensive Cancer Network (NCCN) notes that oral cavity squamous cell carcinomas (OSCC) treated by either radiation therapy or surgery result in similar survival rates, it is recommended that surgery be the main treatment for cancers localized primarily in the oral cavity, and that multimodality treatments be performed for advanced-stage cancers. It has been reported that negative surgical margins and an adequate number of resected lymph nodes correlate to better outcomes [13]. This level of surgical experience and skill as well as pathology expertise are more likely to be found in academic or high-volume treatment facilities. Various studies have addressed facility type or volume and the travel distance required for access [11,14]. For instance, Xia et al. found that longer travel distances were associated with improved survival in bladder cancer patients and that those patients traveling farther predominantly used high volume facilities [11]. Carey et al. similarly found that head and neck cancer patients traveled farther to access academic facilities [14].

For late-stage oral cancers, it is imperative that patients receive the appropriate treatment, but many may not because of discrepancies in treatment plan based on the distance traveled for care. David et al. concluded that, for patients with locally advanced head and neck cancer, facility type, and volume were independently associated with survival [15]. Graboyes et al. [12] noted that “the prevalence of long travel distances for treatment by patients with head and neck squamous cell carcinoma (HNSCC), and the effect of travel distance on overall survival (OS), remains unknown.” Our study’s objective was to examine the correlation between travel distance for therapy and demographic, pathological, and treatment modality, in relation to the overall survival rate of patients with advanced oral cancer.

## 2. Materials and Methods

### 2.1. Study Design

The retrospective analysis of survival outcomes and distance traveled used information extracted from the NCDB. The National Cancer Database is “a nationwide (USA) oncology outcomes database” which captures data from >1500 American College of Surgeons Commission on Cancer accredited oncology programs across the US and Puerto Rico. Approximately 72% of newly diagnosed cancer cases are reported to NCDB by the diagnosing institutions [16].

From NCDB (PUF 2016), 11,121 subjects were extracted for this study, all diagnosed with late-stage squamous cell carcinoma in oral subsites (AJCC 7 T3 and T4) from 2010 to 2016. Mucosal lip cancers were not included. Our dataset included all T3 and T4 cancers, including T4b. Patients with distant metastases were excluded. All patients in the cohort were newly diagnosed and treated with curative intent. The average length of follow-up was 41.2 months (0.0–95.1).

Variables chosen for analyses included demographics, socioeconomic factors, tumor stage and pathologic features, treatment, and facility type as defined in NCDB. Income categories used were specified in NCDB PUF 2016.

Race was designated in NCDB as American Indian or Alaska Native, Asian or Pacific Islander, Black, White or other (self-identified) or unknown. Data for non-white and non-Black patient groups in our study cohort were insufficient to include independently in the analyses with the other variables examined. Therefore, only data for Black and White patients were included in our analyses [17].

There is substantial literature on the impact of facility type and volume on overall cancer patient survival [14,15]. We considered the impact of travel distance on the choice of facility. NCDB is a program of the Commission on Cancer (CoC) which defines cancer program categories based on type and case volume [16]. Facilities in our cohort included Community Cancer Programs (100–500 annual cases), Comprehensive Community Cancer Programs (>500 annual cases), and Academic Comprehensive Cancer Programs (>500 annual cases, medical education, including NCI Comprehensive Cancer Centers). We evaluated Community and Comprehensive Community Cancer Programs together as ‘Community Programs.’

Travel distances were split into quartiles and were assessed for demographic characteristics, pathology, and treatment plan, among other variables. Access was authorized under the approved protocol of Dr. Anurag K. Singh.

### 2.2. Statistical Analysis

Patient demographic and clinical characteristics were summarized by stage at diagnosis and treatment using means, medians, and standard deviations for continuous variables and frequencies and cumulative frequencies for categorical data. Comparisons were made using the Kruskal–Wallis test for continuous variables or Pearson chi-square tests for categorical variables. Overall survival (OS) was summarized by treatment, facility type and distance traveled for treatment using standard Kaplan–Meier methods, where estimates of the median OS were obtained with 95% confidence intervals (CIs). The primary endpoint of the study was to estimate OS, defined by the time from diagnosis to death due to any cause. Comparisons were made using the log-rank test. Univariate and multivariable Cox proportional hazard models were used to estimate the effects of different covariates on OS in study participants. Result estimates were expressed as hazard ratios (HRs) with 95% confidence intervals. All analyses were conducted in SAS v9.4 (Cary, NC, USA) at a significance level of 0.05. We chose a conservative approach to missing data and excluded any subject with missing data in our selected variables. This resulted in only 8070 subjects in our multivariate cohort with the loss mostly attributable to missing facility and pathology data. Despite the loss of subjects, proportional hazard model assumptions were met. The multivariate model was examined for multicollinearity by eigenvalues and VIF. All model assumptions were validated visually and with goodness of fit statistics.

## 3. Results

### 3.1. Cohort Characteristics and Distance Traveled

Our late-stage oral cancer cohort included 11,121 patients with a mean age 62.2 (SD 13.2 years), 63.1% male and 86% White. The distance traveled (miles) was measured between the centroid of the patient’s zip code and the zip code of the treatment facility. We designated D1–4 as the four quartiles of distance traveled (D1 < 7.25, D2 7.25–17.6, D3 17.6–46.5, D4 > 46.5 miles).

Patient cohorts defined by travel distance were assessed for demographic variables including race, income, insurance, and language spoken (Table 1). Black and Spanish-speaking patients, and those in urban settings, were more likely to travel the shortest distance for care (first quartile—D1); White and rural patients were more likely to be in D4. Patients living in rural areas were 18× more likely to be in D4 (*p* < 0.001), while those in urban areas were more likely to be in D1. Patients with both the lowest (less than USD 38,000/yr) and highest (more than USD 63,000/yr) median household incomes were more likely to be in D1, while patients with an income of USD 38,000–47,999 were more likely in D4. Comorbidity and the type of insurance were not associated with distance traveled for care.

Academic/research facilities were the preferred site of treatment for patients in D4 (~2x, *p* < 0.001, Table 1) versus community centers for those in D1. Patients who received care at a community cancer program (considering Community Cancer Programs and Comprehensive Community Cancer Programs together) were ~3 times more likely to be in D1 compared to D4 (*p* < 0.001).

### 3.2. Cancer Subsite and Treatment Type

Survival outcomes are also associated with the anatomical location of a tumor [18]. Therefore, we looked at several oral subsites (Table 2) to determine possible correlations between tumor location and distance traveled for treatment. We found a significant travel distance difference among patients diagnosed with oral tongue, gingiva, and alveolar cancers. Patients with oral tongue cancer, the most common diagnosis within the cohort, were more likely to be in D1, while those with gingiva and alveolar cancer, the third most common, were more likely to be in D4. There was no difference in distance traveled for patients diagnosed with any other recorded subsites.

NCCN guidelines recommend multimodality treatment for late-stage oral cancer [19]. Surgery- and radiation-based plans are the predominant modalities to treat oral cancer with curative intent. We found a statistically significant difference in travel distance for patients who received surgery- versus radiation-based modalities. Among patients in the fourth distance quartile, 92.1% received surgery as primary management. Surgery plus radiation and surgery plus radiation and chemotherapy were not statistically different across distances traveled.

### 3.3. Pathology

Among patients who underwent surgical-based management, we evaluated various pathological parameters to assess the quality of surgical resection. Specifically, positive margins correlate with tumor recurrence and decreased survival rate [13,20,21]. Patients with grossly and microscopically negative surgical margins differed based on distance traveled with 80.4% of patients in D4, versus 65.8% of those in D1 (Table 2).

In addition, regional lymph node metastasis predicts aggressive tumor behavior and portends poor locoregional control and survival outcome [22]. NCCN recommends pathologic review of lymph nodes in late-stage cancers as proper management of the regional lymphatic basin. Comprehensiveness of neck dissection is important in this regard. It has been reported that both higher number of metastatic lymph nodes as well as higher lymph node ratio (regional nodes positive: regional nodes examined) predicts poorer prognosis [23,24,25,26]. Although positive nodes were identified in similar proportions for those in D1 and D4, patients in the fourth quartile of distance traveled had a higher total number of lymph nodes examined (D4, mean = 33.7; D1, mean = 25.6), indicating more comprehensive neck dissection (Table 2).

For a significant number of late-stage oral cancer patients in D1, the neck was not even addressed. Lymph nodal basin was not examined in 24.1% of the D1 surgical cohort compared to 8.7% in the D4 surgical cohort. This was also indicated by a three-fold difference in pNx patients between D1 and D4 cohorts (D1: 16.4%, D4: 5.6%), indicating that the nodes could not be assessed (Table 2).

### 3.4. Distance Traveled for the Treatment and Treatment Facility

We also noted that patients in D3 and D4 were more likely to travel for treatment at academic/research institutions. By traveling to such facilities, patients in the longest travel distance cohort (D4) are more likely to receive treatment with adequate and thorough surgical resection (Table 1 and Table 2).

**Figure 1:**
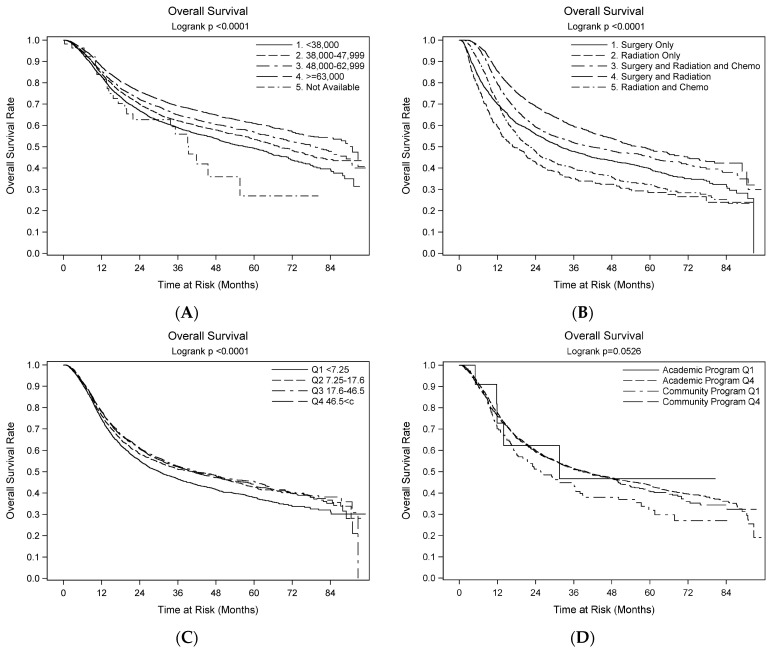
Overall survival in late-stage oral cancer. The Kaplan–Meier plots show the statistically significant impacts of household income (**A**), treatment plan (**B**), distance traveled for treatment (**C**), and type of treatment setting (**D**).

We found that patients who traveled the farthest (D4) for care had the best outcome whether at Community or Academic Centers—Academic Q4 (78.3 months) and Community Program Q4 (72.6 months) (Figure 1D). Patients treated at a nearby Community Q1 (D1) (median survival 50.2 months) were more likely to have the lowest 3-year and 5-year survival rates (Figure 1D). For clarity of presentation, we only display Q1 (D1) and Q4 (D4) for each category in this figure. Patients who traveled a longer distance, therefore, were more likely doing so for treatment at an academic program, explaining many of the differences in the treatment choices and outcomes.

### 3.5. Survival Outcomes—Univariate

Univariate analysis of our data showed that patients who had positive margins and positive nodes fared statistically worse than patients who had negative margins and negative lymph nodes (*p* < 0.001). This is in accordance with reported data [27]. Similarly, patients who either had regional nodes positive (RNP) or unknown in comparison to patients with “no nodes positive” had reduced overall survival (*p* < 0.001) (Table 3).

Patients with multimodality treatment had better overall survival than those with a single modality (*p* < 0.001) (Table 3, Figure 1B). For multimodal treatments, a combination of surgery and radiation had the highest 3-year and 5-year patient survival rates, followed by combined surgery, radiation, and chemotherapy. Radiation and chemotherapy had the lowest survival rate among the three combination treatments. Non-surgical treatment was more likely to be given to patients in the D1 cohort compared to D4 (Table 2). Additionally, patients who received treatment at an academic or research center typically traveled farther to receive such treatment than those in D1 (Table 2) and fared better (Figure 1D). In summary, people who traveled farther to receive treatment did so to access an academic or research facility. Most likely, treatment at these centers was surgically based. There was no significant survival difference between patients from urban and rural areas, or between Black and White patients (Table 3).

### 3.6. Survival Outcomes—Multivariate

The statistical findings between univariate and multivariate analyses remained consistent for several variables, denoting their strong correlation to patient survival in the cohort. Patients with negative margins and no nodes positive had better survival outcomes than patients with positive margins or nodes positive (*p* < 0.001), respectively (Table 4). Patients with regional node positivity unknown (pNx) had poorer survival than patients with no nodes positive (*p* < 0.001), indicating the importance of addressing the regional lymph node basin (Table 4). As noted above, the D1 cohort was three-fold more likely to have pNx designation than the D4 group (Table 2).

Using multivariate analysis, patients receiving multimodality therapy had better survival than patients who received surgery only (*p* < 0.001). We also noted that patients with surgery-based therapy had a higher median survival than patients with non-surgical intervention (Table 4, Figure 1B).

Patients in D2, D3 and D4 statistically had better survival than D1 on multivariate analysis (*p* < 0.006) (Table 4, Figure 1C).

Due to exclusion of all subjects with missing data in relevant variables (primarily facility type), our multivariable cohort was smaller than our univariate cohort and the two cohorts did show statistically significant differences in some variables (Table S1). This sensitivity analysis indicates that the results from the multivariable analysis may not be generalizable to the whole population; however, the conclusions drawn from the full cohort (univariate) remained significant in the smaller cohort (multivariable). This subject loss represents a limitation of our study, but the consistency between analyses provides confidence in the validity of our conclusions. Sensitivity analysis found that the majority of discrepancies between the univariate and multivariate outcomes were in effect size, not direction or significance (Table S1).

## 4. Discussion

In our retrospective study, we report outcomes for patients with late-stage oral cancers and association with the distance traveled for treatment. Further analysis evaluated differences in the type of treatment facility and treatment received as a function of distance traveled for care. Management of late-stage oral cancer can be divided into surgical-based and non-surgical-based approaches. For resectable late-stage cancers, NCCN guidelines favor surgical-based modalities [19]. Disparities in oral cavity squamous cell cancers diagnosis and treatment, in part, arise from delayed cancer screening which can result in some patients presenting with late-stage cancers at the time of primary diagnosis [26,28]. Additionally, the longer the travel time to a provider, the more likely a patient is to be diagnosed with advanced stage OSCC, especially among low-income patients [29]. Treatment location and travel distance, therefore, have the potential to further stratify access to care for those with late-stage oral cancer. In a review of the literature, Ambroggi et al. reported travel distance as an important factor hindering access to appropriate and timely cancer care [9]. Similarly, Payne et al. reported on psychological distress and reduced treatment compliance associated with longer travel distance [30].

The negative impact of travel distance for cancer treatment cannot be denied. It is, however, equally important to seek treatment at a facility adequately equipped to provide such care. Treatment at low volume and non-academic facilities has been reported to be associated with poorer survival outcomes [31]. Vetterlein et al. examined the impact of travel distance on the management of prostate cancer and showed that men who traveled longer distances had better outcomes influenced by treatment modality and facility-level factors [32]. Several other studies have also reported Cox proportional hazard models demonstrating improved overall survival with greater distance traveled for care [10,11,12]. To date, the impact of travel distance on the survival outcome of late-stage oral cavity cancer has not been comprehensively evaluated [12,33]. Our study is the first to report an association of travel distance with treatment modality, adequacy of treatment, and facility type as it pertains to the management of late-stage oral cancer. We also reported additional demographic factors that may influence such travel.

In our study, Black, Spanish-speaking, and low-income patients were more likely to seek care locally than travel farther. Patients in the highest quartile of median household income were also less likely to travel. In terms of overall survival, patients with median income of less than USD 38,000 had the poorest 3-year, 5-year and median survival rate compared to all other income brackets, except for patients whose financial information was unavailable. Weng et al. also reported that Black patients are less likely to be recommended for surgery-based therapies to treat OSCC than their White counterparts [34].

High volume academic centers provide specialized care for advanced cases concordant with NCCN guidelines for treatment [33]. David et al. suggested that factors driving improved outcomes include “access to ancillary services such as nutrition, palliative care, swallow therapy and oncologic psychiatry that increase the probability of treatment completion, minimize the likelihood of treatment interruptions and mitigate morbidity” [15]. Our results have implications for patient counseling on benefits of seeking healthcare in a multidisciplinary facility and the value of a longer travel distance to do so.

Patients who traveled greater distances were more likely to receive surgical-based treatment with better survival. The adequacy of surgical resection was significantly superior in the D4 cohort, with better rates of negative margins and comprehensive neck dissection. This reflects the consequence of centralization of complex cancer care in the United States [35]. People in the D4 cohort generally traveled to receive treatment at academic facilities. While travel distance may pose a significant barrier to access quality care, high-volume or academic facilities should result in population-level improvement in cancer outcome, as is seen in our current study.

We also noted that patients with both nodes positive and nodes “unknown” (pNx) fared statistically worse compared to patients with no nodes positive (*p* < 0.001). Osarogiabon and Yu determined that when diagnosed with non-small cell lung cancer, patients with pN0 had a two-fold higher median overall survival compared to patients with pNx [36]. In our study, we saw that patients who received treatment close to home, which were often community centers, were more likely to be designated with a pNx status. This underscores that where patients go for care may influence whether and how well nodes were examined, especially because low-volume facilities may lack specialized physicians who can perform ablative and reconstructive procedures often needed for late-stage oral cavity cancers. While NCDB provided margin, node, and lymphovascular invasion data, it did not provide enough detail of other pathological features, such as perineural invasion, and extranodal extension, that may influence treatment choices and represent confounding variables.

Travel distance was correlated with both the selected facility type and the median survival for patients in our cohort. A greater distance traveled was associated with receiving care at an academic program and having a better median survival. Although not captured directly with the NCDB, there are also the considerations of social support, physical capacity and transportation access that may interfere with travel and hinder treatment compliance and overall health outcomes. We acknowledge that long-distance travel can present as a significant social and financial burden to patients; however, patients who traveled farther were more likely to receive treatment according to best clinical practices and to have better survival outcomes.

When considering distance traveled, however, quartile designations may obscure key nuances in pinpointing OSCC disparities in survival. The fourth distance quartile ranged from 46.6 to 2815.2 miles (mean ± SD: 134.1 ± 200.3), suggesting that a variety of transportation options were used. It is important to keep in mind that ten miles by car can be a drastically different experience by bus, which presents a host of challenges in access to care. Since both low-income and high-income patients were more likely to seek treatment in D1, evaluating patient distribution within a given zip code might provide a more granular perspective on the various socioeconomic factors impacting survival. It therefore may be informative to look at travel time and mode, considering both same-day and overnight travel. Fewer than 20% of the patients in this cohort were managed at multiple facilities according to the NCDB multi-source variable. However, the nature of the care provided at these multiple facilities was not specified [33,37,38]. For example, patients may travel a greater distance for surgery then receive adjuvant treatment closer to home.

For the purpose of this study, we focused on late-stage oral cancer, but this introduces some confounding factors in that a particular cancer may be considered resectable in one clinical center but not another. NCDB provides distinction between specific facility types, but physician skill and ancillary supports will still vary within these categories. Limitations have been mentioned as part of our discussion but include the retrospective nature of our evaluation, selection bias between our cohorts, lack of longitudinal treatment data, lack of clinically relevant endpoints and underrepresentation of specific populations.

Despite the known biases and confounders inherent in this type of study, we consider it well established that travel distance adds to the burden of cancer treatment for patients, but our data demonstrate that traveling to receive care at an academic clinical center can provide enhanced overall survival. As a matter of public health, this should be noted by referring physicians.

## 5. Conclusions

Our analyses identified significant associations of travel distance with care received, facility type and outcomes. These data support the value of patient counseling and referral to optimize their clinical management and consideration of socioeconomic factors.

## Figures and Tables

**Table 1:** Late-stage oral cancer descriptive statistics by distance—demographic information.

		D1 <7.25 miles	D2 7.25–17.6	D3 17.6–46.5	D4 >46.5 miles	Total	*p* Value
Overall	N (%)	2764 (24.9%)	2714 (24.2%)	2715 (24.5%)	2910 (26.2%)	11,121 (100%)	
Age	Mean ± SD	62.5 ± 13.4	62.5 ± 13.2	62.0 ± 13.0	62.0 ± 13.1	62.2 ± 13.2	0.490
Sex	Male	1736 (62.8%)	1689 (62.2%)	1716 (62.2%)	1869 (64.2%)	7019 (63.1%)	0.462
	Female	1028 (37.2%)	1025 (37.8%)	999 (36.8%)	1041 (35.8%)	4102 (36.9%)	
Race	White	2183 (79.0%)	2297 (84.6%)	2414 (88.8%)	2652 (91.1%)	9559 (86.0%)	<0.001
	Black	370 (13.4%)	206 (7.7%)	147 (5.4%)	175 (6.0%)	904 (8.1%)	
Spanish-speaking	No	2473 (89.5%)	2499 (92.1%)	2566 (94.5%)	2777 (95.4%)	10,330 (92.9%)	<0.001
	Yes	223 (8.1%)	155 (5.7%)	101 (3.7%)	81 (2.8%)	563 (5.1%)	
Median Household Income	<$38,000	685 (24.8%)	258 (9.5%)	372 (13.7%)	758 (26.0%)	2075 (18.7%)	<0.001
	$38,000–$47,999	687 (24.9%)	454 (16.7%)	712 (26.2%)	1060 (36.4%)	2913 (26.2%)	
	$48,000–$62,999	653 (23.6%)	855 (31.5%)	745 (27.4%)	715 (24.6%)	2968 (26.2%)	
	≥$63,000	736 (26.6%)	1141 (42.1%)	885 (32.6%)	371 (12.7%)	3135 (28.2%)	
	Not Available	3 (0.1%)	5 (0.2%)	1 (0.0%)	6 (0.2%)	30 (0.3%)	
Insurance	No Insurance	181 (6.5%)	162 (6.0%)	163 (6.0%)	167 (5.7%)	673 (6.1%)	0.002
	Private Insurance	937 (33.9%)	1074 (39.6%)	1406 (38.5%)	1054 (36.2%)	4118 (37.0%)	
	Government Insurance	1605% (58.1%)	1435 (52.9%)	1467 (54.0%)	1635 (56.2%)	6153 (55.3%)	
Urban	Rural	33 (1.2%)	29 (1.1%)	119 (4.5%)	611 (21.6%)	793 (7.3%)	<0.001
	Urban	2680 (98.8%)	2637 (98.9%)	2539 (95.5%)	2217 (78.4%)	10,080 (92.7%)	
Cancer Stage	Stage III	731 (26.4%)	691 (25.5%)	688 (25.3%)	658 (22.6%)	2772 (24.9%)	0.006
	Stage IV	2033 (73.6%)	2023 (74.5%)	2027 (74.7%)	2252 (77.4%)	8349 (75.1%)	
Facility	Community Cancer Program	989 (44.4%)	785 (35.4%)	633 (27.2%)	293 (11.3%)	2702 (28.8%)	<0.001
	Academic/Research Program	1237 (55.6%)	1434 (64.6%)	1692 (72.8%)	2302 (88.7%)	6680 (71.2%)	
Distance	Mean ± SD	3.8 ± 1.9	11.0 ± 3.0	29.3 ± 9.3	134.1 ± 200.3	46.1 ± 115.6	

**Table 2:** Late-stage oral cancer descriptive statistics by distance—subsite, treatment, and tumor.

		D1 <7.25 miles	D2 7.25–17.6	D3 17.6–46.5	D4 >46.5 miles	Total	*p* Value
Site Recorded	Oral tongue	1219 (44.1%)	1249 (46.0%)	1131 (41.7%)	1086 (37.3%)	4696 (42.2%)	<0.001
	Gingiva and alveolus	383 (13.9%)	412 (15.2%)	470 (17.3%)	602 (37.3%)	1869 (16.8%)	
	Floor of mouth	525 (19.0%)	445 (16.4%)	459 (16.9%)	555 (19.1%)	1988 (17.9%)	
	Hard palate	122 (4.4%)	92 (3.4%)	100 (3.7%)	103 (3.5%)	417 (3.7%)	
	Buccal mucosa	213 (7.7%)	217 (8.0%)	224 (8.3%)	217 (7.5%)	871 (7.8%)	
	Retromolar trigone	169 (6.1%)	182 (6.7%)	205 (7.6%)	208 (7.1%)	765 (6.9%)	
	Other and unspecified mouth	133 (4.8%)	117 (4.3%)	126 (4.6%)	139 (4.8%)	515 (4.6%)	
Chemo	No	1422 (51.4%)	1465 (54.0%)	1584 (58.3%)	1878 (64.5%)	6356 (57.2%)	<0.001
	Yes	1342 (48.6%)	1249 (46.0%)	1131 (41.7%)	1032 (35.5%)	4765 (42.8%)	
Radiation	No	498 (18.0%)	568 (20.9%)	400 (14.7%)	231 (7.9%)	1866 (16.8%)	<0.001
	Yes	2266 (82.0%)	2146 (79.1%)	2315 (85.3%)	2679 (92.1%)	9255 (83.2%)	
Surgery	No	684 (24.7%)	548 (79.8%)	400 (14.7%)	231 (7.9%)	1866 (16.8%)	<0.001
	Yes	2080 (75.3%)	2166 (79.8%)	2315 (85.3%)	2679 (92.1%)	9255 (83.2%)	
Treatment	Surgery Only	498 (18.0%)	568 (20.9%)	702 (25.9%)	1017 (34.9%)	2787 (25.1%)	<0.001
	Radiation Only	206 (7.5%)	168 (6.2%)	105 (3.9%)	72 (2.5%)	552 (5.0%)	
	Surgery Radiation & Chemo	864 (31.3%)	869 (32.0%)	836 (30.8%)	873 (30.0%)	3451 (31.0%)	
	Surgery and Radiation	718 (26.0%)	729 (26.9%)	777 (28.6%)	789 (27.1%)	3017 (27.1%)	
	Radiation and Chemo	478 (17.3%)	380 (14.0%)	295 (10.9%)	158 (5.5%)	1314 (11.8%)	
Reason for no surgery	Surgery of the primary site was performed	2222 (80.4%)	2320 (85.5%)	2426 (89.4%)	2757 (94.7%)	9741 (87.6%)	<0.001
	Surgery was not a part of the planned first course treatment	457 (16.5%)	333 (12.3%)	251 (9.2%)	118 (4.1%)	1161 (10.4%)	
	Surgery was contraindicated due to patient risk factors	34 (1.2%)	29 (1.1%)	16 (0.6%)	12 (0.4%)	91 (0.8%)	
TNM Path N (%)	0	507 (20.0%)	533 (20.9%)	660 (25.5%)	787 (27.9%)	2489 (23.7%)	<0.001
	1	581 (23.0%)	600 (23.5%)	564 (21.8%)	656 (23.2%)	2405 (22.5%)	
	2	1006 (39.8%)	1080 (42.3%)	1093 (42.2%)	1196 (42.4%)	4382 (41.7%)	
	3	21 (0.8%)	18 (0.7%)	18 (0.7%)	20 (0.7%)	78 (0.7%)	
	X	415 (16.4%)	322 (12.5%)	253 (9.8%)	159 (5.6%)	1150 (10.9%)	
Regional Nodes Examined	Mean ± SD	25.6 ± 23.2	27.9 ± 22.3	30.1 ± 22.5	33.7 ± 22.2	29.0 ± 22.8	<0.001
RNE (cat.)	No nodes examined	666 (24.1%)	487 (17.9%)	392 (14.4%)	254 (8.7%)	1801 (16.2%)	<0.001
	Nodes examined	2084 (75.4%)	2213 (81.5%)	2313 (85.2%)	2648 (91.0%)	9273 (83.4%)	
	Unknown	791 (28.6%)	605 (22.3%)	465 (17.1%)	393 (13.5%)	2256 (20.3%)	
RNE (cat.) Regional Nodes Positive	Mean ± SD	2.5 ± 3.5	2.4 ± 3.5	2.0 ± 3.8	2.3 ± 3.9	2.4 ± 3.7	<0.001
RNP (cat.)	No nodes positive	469 (17.0%)	494 (18.2%)	624 (23.0%)	763 (26.2%)	2352 (21.1%)	<0.001
	Nodes positive	1621 (58.6%)	1724 (63.5%)	1691 (62.3%)	1886 (64.8%)	6936 (62.4%)	
	Unknown	674 (24.4%)	496 (18.3%)	400 (14.7%)	261 (9.0%)	1833 (16.5%)	
RX_SUMM Surgical Margins	all margins grossly & microscopically negative	1820 (65.8%)	1951 (71.9%)	2045 (75.3%)	2340 (80.4%)	8169 (73.5%)	<0.001
	margins positive	161 (5.8%)	155 (5.7%)	160 (5.9%)	197 (6.8%)	675 (6.1%)	

**Table 3:** Univariate model results.

Model	Dependent Variable	Reference Level	Results Estimate (95% CL) *p*-Value
OS	Sex (Female)	(Ref: Male)	0.99 (0.93, 1.06) *p* = 0.837
	Race (Black)	(Ref: White)	1.05 (0.93, 1.19) *p* = 0.410
	Treatment (Radiation only)	(Ref: Surgery Only)	0.61 (0.48, 0.78) *p* ≤ 0.001
	Treatment (Radiation and Chemo)	(Ref: Surgery Only)	0.66 (0.53, 0.83) *p* ≤ 0.001
	Treatment (Surgery and Radiation)	(Ref: Surgery Only)	0.64 (0.59, 0.60) *p* ≤ 0.001
	Treatment (Surgery and Radiation and Chemo)	(Ref: Surgery Only)	0.80 (0.74, 0.86) *p* ≤ 0.001
	Urban (Rural)	(Ref: Urban)	1.07 (0.95, 1.20) *p* = 0.285
	Facility (Academic/Research)	(Ref: Community Cancer Program)	0.98 (0.91, 1.05) *p* = 0.560
	Insurance (Government Insurance)	(Ref: No Insurance)	1.39 (1.20, 1.60) *p* ≤ 0.001
	Insurance (Private Insurance)	(Ref: No Insurance)	0.86 (0.74, 1.35) *p* = 0.059
	Surgical Margins (positive)	(Ref: negative)	1.55 (1.42, 1.69) *p* ≤ 0.001
	Distance (D2 7.25–17.6)	(Ref: D1 < 7.25)	0.87 (0.79, 0.95) *p* = 0.004
	Distance (D3 17.6–46.5)	(Ref: D1 < 7.25)	0.85 (0.78, 0.94) *p* ≤ 0.001
	Distance (D4 46.5 < c)	(Ref: D1 < 7.25)	0.90 (0.82, 0.98) *p* = 0.018
	RNP (Nodes positive)	(Ref: Non nodes positive)	1.87 (1.71, 2.04) *p* ≤ 0.001
	RNP (Unknown)	(Ref: Non nodes positive)	1.63 (1.39, 1.90) *p* ≤ 0.001
	Age		1.02 (1.02, 1.02) *p* ≤ 0.001

**Table 4:** Multivariate model results *.

Model	Dependent Variable	Reference Level	Results Estimate (95% CI) *p*-Value
OS	Sex (Female)	(Ref: Male)	0.91 (0.85, 0.97) *p* = 0.007
	Race (Black)	(Ref: White)	1.05 (0.93, 1.19) *p* = 0.394
	Treatment (Radiation only)	(Ref: Surgery Only)	0.57 (0.45, 0.73) *p* ≤ 0.001
	Treatment (Radiation and Chemo)	(Ref: Surgery Only)	0.63 (0.50, 0.79) *p* ≤ 0.001
	Treatment (Surgery and Radiation)	(Ref: Surgery Only)	0.67 (0.61, 0.63) *p* ≤ 0.001
	Treatment (Surgery, Radiation & Chemo)	(Ref: Surgery Only)	0.78 (0.71, 0.85) *p* ≤ 0.001
	Urban (Rural)	(Ref: Urban)	1.05 (0.93, 1.10) *p* = 0.416
	Facility (Academic/Research)	(Ref: Community Cancer Program)	1.03 (0.95, 1.11) *p* = 0.427
	Insurance (Government Ins.)	(Ref: No Insurance)	1.08 (0.93, 1.19) *p* = 0.328
	Insurance (Private Insurance)	(Ref: No Insurance)	0.82 (0.71, 0.96) *p* = 0.011
	Surgical Margins (positive)	(Ref: negative)	1.51 (1.39, 1.64) *p* ≤ 0.001
	Distance (D2 7.25–17.6)	(Ref: D1 < 7.25)	0.86 (0.78, 0.94) *p* = 0.002
	Distance (D3 17.6–46.5)	(Ref: D1 < 7.25)	0.86 (0.79, 0.95) *p* = 0.002
	Distance (D4 46.5 < c)	(Ref: D1 < 7.25)	0.87 (0.79, 0.96) *p* = 0.006
	RNP (Nodes positive)	(Ref: Non nodes positive)	2.00 (1.82, 2.20) *p* ≤ 0.001
	RNP (Unknown)	(Ref: Non nodes positive)	1.39 (1.18, 1.64) *p* ≤ 0.001
	Age		1.02 (1.02, 1.02) *p* ≤ 0.001

* The adjustment set for multivariate models is provided as Supplemental Table S2.

## Data Availability

The primary dataset (National Cancer Database) is available publicly for investigators associated with Commission on Cancer-accredited programs through the American College of Surgeons (https://www.facs.org/quality-programs/cancer/ncdb, accessed on 21 July 2024).

## Supplementary Materials

The following supporting information can be downloaded at: https://www.mdpi.com/article/10.3390/cancers16152750/s1, Table S1. Demographic Analysis of Missing Data Exclusion. Table S2. Multivariable Cox Hazard Ratio Modeling Adjustment Set.

## References

[1] Siegel R.L., Giaquinto A.N., Jemal A. (2024). Cancer statistics, 2024. CA Cancer J. Clin..

[2] Dhanuthai K., Rojanawatsirivej S., Thosaporn W., Kintarak S., Subarnbhesaj A., Darling M., Kryshtalskyj E., Chiang C.P., Shin H.I., Choi S.Y. (2018). Oral cancer: A multicenter study. Med. Oral Patol. Oral Cir. Bucal.

[3] Osazuwa-Peters N., Adjei Boakye E., Hussaini A.S., Sujijantarat N., Ganesh R.N., Snider M., Thompson D., Varvares M.A. (2017). Characteristics and predictors of oral cancer knowledge in a predominantly African American community. PLoS ONE.

[4] Curtis D.C., Eckhart S.C., Morrow A.C., Sikes L.C., Mridha T. (2020). Demographic and Behavioral Risk Factors for Oral Cancer among Florida Residents. J. Int. Soc. Prev. Community Dent..

[5] Brennan M.T., Treister N.S., Sollecito T.P., Schmidt B.L., Patton L.L., Yang Y., Lin A., Elting L.S., Hodges J.S., Lalla R.V. (2021). Epidemiologic factors in patients with advanced head and neck cancer treated with radiation therapy. Head Neck.

[6] Smith D.K., Murphy B.A. (2019). Lower levels of education and household income mediate lower dental care utilization among survivors of early life cancers. Prev. Med. Rep..

[7] Agarwal P., Agrawal R.R., Jones E.A., Devaiah A.K. (2020). Social Determinants of Health and Oral Cavity Cancer Treatment and Survival: A Competing Risk Analysis. Laryngoscope.

[8] Singh A., Peres M.A., Watt R.G. (2019). The Relationship between Income and Oral Health: A Critical Review. J. Dent. Res..

[9] Ambroggi M., Biasini C., Del Giovane C., Fornari F., Cavanna L. (2015). Distance as a Barrier to Cancer Diagnosis and Treatment: Review of the Literature. Oncologist.

[10] Lamont E.B., Hayreh D., Pickett K.E., Dignam J.J., List M.A., Stenson K.M., Haraf D.J., Brockstein B.E., Sellergren S.A., Vokes E.E. (2003). Is patient travel distance associated with survival on phase II clinical trials in oncology?. J. Natl. Cancer Inst..

[11] Xia L., Taylor B.L., Mamtani R., Christodouleas J.P., Guzzo T.J. (2018). Associations Between Travel Distance, Hospital Volume, and Outcomes Following Radical Cystectomy in Patients With Muscle-invasive Bladder Cancer. Urology.

[12] Graboyes E.M., Ellis M.A., Li H., Kaczmar J.M., Sharma A.K., Lentsch E.J., Day T.A., Hughes Halbert C. (2018). Racial and Ethnic Disparities in Travel for Head and Neck Cancer Treatment and the Impact of Travel Distance on Survival. Cancer.

[13] Mannelli G., Comini L.V., Piazza C. (2019). Surgical margins in oral squamous cell cancer: Intraoperative evaluation and prognostic impact. Curr. Opin. Otolaryngol. Head Neck Surg..

[14] Carey R.M., Fathy R., Shah R.R., Rajasekaran K., Cannady S.B., Newman J.G., Ibrahim S.A., Brant J.A. (2020). Association of Type of Treatment Facility With Overall Survival After a Diagnosis of Head and Neck Cancer. JAMA Netw. Open.

[15] David J.M., Ho A.S., Luu M., Yoshida E.J., Kim S., Mita A.C., Scher K.S., Shiao S.L., Tighiouart M., Zumsteg Z.S. (2017). Treatment at high-volume facilities and academic centers is independently associated with improved survival in patients with locally advanced head and neck cancer. Cancer.

[16] The American College of Surgeons About the National Cancer Database. www.facs.org/quality-programs/cancer-programs/national-cancer-database/about/.

[17] Flanagin A., Frey T., Christiansen S.L., AMA Manual of Style Committee (2021). Updated Guidance on the Reporting of Race and Ethnicity in Medical and Science Journals. JAMA.

[18] Moro J.D.S., Maroneze M.C., Ardenghi T.M., Barin L.M., Danesi C.C. (2018). Oral and oropharyngeal cancer: Epidemiology and survival analysis. Einstein.

[19] Pfister D.G., Spencer S., Adelstein D., Adkins D., Anzai Y., Brizel D.M., Bruce J.Y., Busse P.M., Caudell J.J., Cmelak A.J. (2020). Head and Neck Cancers, Version 2.2020, NCCN Clinical Practice Guidelines in Oncology. J. Natl. Compr. Cancer Netw..

[20] Williams M.D. (2016). Determining Adequate Margins in Head and Neck Cancers: Practice and Continued Challenges. Curr. Oncol. Rep..

[21] Otsuru M., Hasegawa T., Yamakawa N., Okura M., Yamada S.I., Hirai E., Inomata T., Saito H., Miura K.I., Furukawa K. (2023). A Multicenter Study on the Effect of Margin Distance on Survival and Local Control in Stage 1–2 Squamous Cell Carcinoma of the Tongue. Ann. Surg. Oncol..

[22] Gogna S., Kashyap S., Gupta N. (2024). Neck Cancer Resection and Dissection. StatPearls.

[23] Ho A.S., Kim S., Tighiouart M., Gudino C., Mita A., Scher K.S., Laury A., Prasad R., Shiao S.L., Ali N. (2018). Association of Quantitative Metastatic Lymph Node Burden With Survival in Hypopharyngeal and Laryngeal Cancer. JAMA Oncol..

[24] de Ridder M., Marres C.C., Smeele L.E., van den Brekel M.W., Hauptmann M., Balm A.J., van Velthuysen M.L. (2016). A critical evaluation of lymph node ratio in head and neck cancer. Virchows Arch..

[25] Lee H., Roh J.L., Cho K.J., Choi S.H., Nam S.Y., Kim S.Y. (2019). Number of positive lymph nodes better predicts survival for oral cavity cancer. J. Surg. Oncol..

[26] Adrien J., Bertolus C., Gambotti L., Mallet A., Baujat B. (2014). Why are head and neck squamous cell carcinoma diagnosed so late? Influence of health care disparities and socio-economic factors. Oral Oncol..

[27] Zanoni D.K., Montero P.H., Migliacci J.C., Shah J.P., Wong R.J., Ganly I., Patel S.G. (2019). Survival outcomes after treatment of cancer of the oral cavity (1985–2015). Oral Oncol..

[28] Villa A., Stock S., Aboalela A., Lerman M.A., Woo S.B., Sonis S.T., Treister N.S. (2015). Oral Medicine referrals at a hospital-based practice in the United States. Oral. Surg. Oral. Med. Oral. Pathol. Oral Radiol..

[29] Farquhar D.R., Masood M.M., Lenze N.R., McDaniel P., Mazul A., Sheth S., Zanation A.M., Hackman T.G., Weissler M., Zevallos J.P. (2019). Travel time to provider is associated with advanced stage at diagnosis among low income head and neck squamous cell carcinoma patients in North Carolina. Oral Oncol..

[30] Payne S., Jarrett N., Jeffs D. (2000). The impact of travel on cancer patients’ experiences of treatment: A literature review. Eur. J. Cancer Care.

[31] Luryi A.L., Chen M.M., Mehra S., Roman S.A., Sosa J.A., Judson B.L. (2015). Treatment Factors Associated With Survival in Early-Stage Oral Cavity Cancer: Analysis of 6830 Cases From the National Cancer Data Base. JAMA Otolaryngol. Head Neck Surg..

[32] Vetterlein M.W., Loppenberg B., Karabon P., Dalela D., Jindal T., Sood A., Chun F.K., Trinh Q.D., Menon M., Abdollah F. (2017). Impact of travel distance to the treatment facility on overall mortality in US patients with prostate cancer. Cancer.

[33] Ringstrom M.J., Christian J., Bush M.L., Levy J.E., Huang B., Gal T.J. (2018). Travel distance: Impact on stage of presentation and treatment choices in head and neck cancer. Am. J. Otolaryngol..

[34] Weng Y., Korte J.E. (2012). Racial disparities in being recommended to surgery for oral and oropharyngeal cancer in the United States. Community Dent. Oral Epidemiol..

[35] Stitzenberg K.B., Sigurdson E.R., Egleston B.L., Starkey R.B., Meropol N.J. (2009). Centralization of cancer surgery: Implications for patient access to optimal care. J. Clin. Oncol..

[36] Osarogiagbon R.U., Yu X. (2013). Nonexamination of lymph nodes and survival after resection of non-small cell lung cancer. Ann. Thorac. Surg..

[37] Sullivan C.B., Al-Qurayshi Z., Anderson C.M., Seaman A.T., Pagedar N.A. (2021). Factors Associated With the Choice of Radiation Therapy Treatment Facility in Head and Neck Cancer. Laryngoscope.

[38] Tassone P., Topf M.C., Dooley L., Galloway T., Biedermann G., Trendle M. (2023). Going Off Guidelines: An NCDB Analysis of Missed Adjuvant Therapy Among Surgically Treated Oral Cavity Cancer. Otolaryngol. Head Neck Surg..

