# Supplementary material for: Retrospective Cohort Study on the Impact of Travel Distance on Late-Stage Oral Cancer Treatment and Outcomes: An NCDB Analysis
Source: Cancers (Basel). 2024 Aug 2;16(15):2750. doi: 10.3390/cancers16152750 (PMC11311623; doi:10.3390/cancers16152750)
Supplement: Supplementary file 1 [file cancers-16-02750-s001.zip › Harris et al Supplemental Table S1 080124.pdf]

Harris et al Supplemental Table S1. Demographic Analysis of Missing Data Exclusion.

		Missing	Non-Missing	Total	Pval
Overall Cnt	N	3051 (27.4)	8070 (72.6)	11,121 (100%)	
Age	Mean/StdErr	59.8/0.3	63.1/0.1	62.2/0.1	<0.001
	Median/Min/Max	60.0/19.0/90.0	62.0/25.0/90.0	62.0/19.0/90.0	
Age Group	19-69	2184 (71.6%)	5741 (71.1%)	7925 (71.3%)	0.645
	70+	867 (28.4%)	2329 (28.9%)	3196 (28.7%)	
Sex	Male	1957 (64.1%)	5062 (62.7%)	7019 (63.1%)	0.167
	Female	1094 (35.9%)	3008 (37.3%)	4102 (36.9%)	
Race	White	2594 (85.0%)	6965 (86.3%)	9559 (86.0%)	0.036
	Black	288 (9.4%)	616 (7.6%)	904 (8.1%)	
	NBPoC	15 (0.5%)	21 (0.3%)	36 (0.3%)	
	Unknown	13 (0.4%)	35 (0.4%)	48 (0.4%)	
Race Ethnicity	White non-Hispanic	2381 (78.0%)	6496 (80.5%)	8877 (79.8%)	<0.001
	Black non-Hispanic	280 (9.2%)	601 (7.4%)	881 (7.9%)	
	Hispanic	163 (5.3%)	400 (5.0%)	563 (5.1%)	
	Other	133 (4.4%)	397 (4.9%)	530 (4.8%)	
	Unknown	94 (3.1%)	176 (2.2%)	270 (2.4%)	
Spanish	No	2806 (92.0%)	7524 (93.2%)	10,330 (92.9%)	0.009
	Yes	163 (5.3%)	400 (5.0%)	563 (5.1%)	
	Unknown	82 (2.7%)	146 (1.8%)	228 (2.1%)	
MEDIAN_HOUSEHOLD_INCOME	<38000	579 (19.0%)	1496 (18.5%)	2075 (18.7%)	<0.001
	38000-47999	745 (24.4%)	2168 (26.9%)	2913 (26.2%)	
	48000-62999	841 (27.6%)	2127 (26.4%)	2968 (26.7%)	
	>=63000	866 (28.4%)	2269 (28.1%)	3135 (28.2%)	
	Not Available	20 (0.7%)	10 (0.1%)	30 (0.3%)	
Insurance	No Insurance	208 (6.8%)	465 (5.8%)	673 (6.1%)	0.207
	Private Insurance	1111 (36.4%)	3006 (37.2%)	4117 (37.0%)	
	Government Insurance	1682 (55.1%)	4471 (55.4%)	6153 (55.3%)	
	Insurance Status Unknown	50 (1.6%)	128 (1.6%)	178 (1.6%)	
Distance	Mean/StdErr	36.4/2.1	49.8/1.3	46.1/1.1	<0.001
	Median/Min/Max	12.4/0.1/2561.3	21.3/0.1/2815.2	18.1/0.1/2815.2	
Distance Group	Q1 <7.25	1001 (33.0%)	1763 (21.8%)	2764 (24.9%)	<0.001
	Q2 7.25-17.6	840 (27.7%)	1874 (23.2%)	2714 (24.4%)	
	Q3 17.6-46.5	680 (22.4%)	2035 (25.2%)	2715 (24.5%)	
	Q4 46.5<c	512 (16.9%)	2398 (29.7%)	2910 (26.2%)	
ANALYTIC_STAGE_GROUP	Stage III	724 (23.7%)	2048 (25.4%)	2772 (24.9%)	0.073
	Stage IV	2327 (76.3%)	6022 (74.6%)	8349 (75.1%)	
grade1	I/II	2354 (77.2%)	6295 (78.0%)	8649 (77.8%)	0.336
	III	697 (22.8%)	1775 (22.0%)	2472 (22.2%)	

YEAR_OF_DIAGNOSIS	2010	472 (15.5%)	989 (12.3%)	1461 (13.1%)	<0.001
	2011	493 (16.2%)	1113 (13.8%)	1606 (14.4%)	
	2012	469 (15.4%)	1274 (15.8%)	1743 (15.7%)	
	2013	546 (17.9%)	1390 (17.2%)	1936 (17.4%)	
	2014	546 (17.9%)	1569 (19.4%)	2115 (19.0%)	
	2015	525 (17.2%)	1735 (21.5%)	2260 (20.3%)	
DX Year grp	2010-2012	1434 (47.0%)	3376 (41.8%)	4810 (43.3%)	<0.001
	2013-2016	1617 (53.0%)	4694 (58.2%)	6311 (56.7%)	
Chemo	No	1255 (41.1%)	5101 (63.2%)	6356 (57.2%)	<0.001
	Yes	1796 (58.9%)	2969 (36.8%)	4765 (42.8%)	
Radiation	No	401 (13.1%)	2386 (29.6%)	2787 (25.1%)	<0.001
	Yes	2650 (86.9%)	5684 (70.4%)	8334 (74.9%)	
Surgery	No	1495 (49.0%)	371 (4.6%)	1866 (16.8%)	<0.001
	Yes	1556 (51.0%)	7699 (95.4%)	9255 (83.2%)	
Treatment	Surgery Only	401 (13.1%)	2386 (29.6%)	2787 (25.1%)	<0.001
	Radiation Only	367 (12.0%)	185 (2.3%)	552 (5.0%)	
	Surgery and Radiation and Chem	668 (21.9%)	2783 (34.5%)	3451 (31.0%)	
	Surgery and Radiation	487 (16.0%)	2530 (31.4%)	3017 (27.1%)	
	Radiation and Chemo	1128 (37.0%)	186 (2.3%)	1314 (11.8%)	
Site recoded	Oral tongue	1443 (47.3%)	3253 (40.3%)	4696 (42.2%)	<0.001
	Gingiva and alveolus	369 (12.1%)	1500 (18.6%)	1869 (16.8%)	
	Floor of mouth	432 (14.2%)	1556 (19.3%)	1988 (17.9%)	
	Hard palate	161 (5.3%)	256 (3.2%)	417 (3.7%)	
	Buccal mucosa	248 (8.1%)	623 (7.7%)	871 (7.8%)	
	Retromolar trigone	244 (8.0%)	521 (6.5%)	765 (6.9%)	
	Other and unspecified mouth	154 (5.0%)	361 (4.5%)	515 (4.6%)	
Histology	SCC	3051 (100.0%)	8070 (100.0%)	11,121 (100.0%)	
MED_INC_QUAR_16	1. <38000	603 (20.2%)	1610 (20.2%)	2213 (20.2%)	0.081
	2. 38000-47999	701 (23.4%)	2040 (25.6%)	2741 (25.0%)	
	3. 48000-62999	728 (24.3%)	1813 (22.8%)	2541 (23.2%)	
	4. >=63000	958 (32.0%)	2499 (31.4%)	3457 (31.6%)	
CDCC_TOTAL_BEST	0	2369 (77.6%)	5920 (73.4%)	8289 (74.5%)	<0.001
	1	497 (16.3%)	1615 (20.0%)	2112 (19.0%)	
	2	119 (3.9%)	374 (4.6%)	493 (4.4%)	
	3	66 (2.2%)	161 (2.0%)	227 (2.0%)	
TUMOR_SIZE	Mean/StdErr	42.6/1.1	38.1/0.4	39.3/0.4	<0.001
	Median/Min/Max	38.0/1.0/990.0	35.0/0.0/990.0	35.0/0.0/990.0	
Facility	Community Cancer Program	602 (45.9%)	2100 (26.0%)	2702 (28.8%)	<0.001
	Academic/Research Program	710 (54.1%)	5970 (74.0%)	6680 (71.2%)	
FACILITY_TYPE_CD	Community Cancer Program	149 (5.7%)	317 (3.9%)	466 (4.4%)	<0.001
	Comprehensive Community Cancer	545 (21.0%)	1780 (22.1%)	2325 (21.8%)	
	Academic/Research Program	816 (31.4%)	5969 (74.0%)	6785 (63.6%)	
	Integrated Network Cancer Prog	1085 (41.8%)		1085 (10.2%)	

FACILITY_LOCATION_CD	New England	174 (6.7%)	337 (4.2%)	511 (4.8%)	<0.001
	Middle Atlantic	321 (12.4%)	1199 (14.9%)	1520 (14.3%)	
	South Atlantic	698 (26.9%)	1632 (20.2%)	2330 (21.9%)	
	East North Central	458 (17.6%)	1548 (19.2%)	2006 (18.8%)	
	East South Central	147 (5.7%)	637 (7.9%)	784 (7.4%)	
	West North Central	147 (5.7%)	865 (10.7%)	1012 (9.5%)	
	West South Central	190 (7.3%)	734 (9.1%)	924 (8.7%)	
	Mountain	186 (7.2%)	256 (3.2%)	442 (4.1%)	
	Pacific	274 (10.6%)	858 (10.6%)	1132 (10.6%)	
PUF_MULT_SOURCE	No	2522 (82.7%)	6495 (80.5%)	9017 (81.1%)	0.009
	Yes	529 (17.3%)	1575 (19.5%)	2104 (18.9%)	
NO_HSD_QUAR_12	>=29%	512 (16.9%)	1464 (18.2%)	1976 (17.8%)	0.023
	20-28.9%	874 (28.8%)	2193 (27.2%)	3067 (27.6%)	
	14-19.9%	957 (31.5%)	2703 (33.5%)	3660 (33.0%)	
	< 14%	691 (22.8%)	1702 (21.1%)	2393 (21.6%)	
TNM_CLIN_STAGE_GROUP	1	112 (3.7%)	457 (5.7%)	569 (5.1%)	<0.001
	2	222 (7.3%)	1039 (12.9%)	1261 (11.3%)	
	3	608 (19.9%)	1560 (19.3%)	2168 (19.5%)	
	4	1972 (64.6%)	4370 (54.2%)	6342 (57.0%)	
	0	7 (0.2%)	15 (0.2%)	22 (0.2%)	
	Other(IS8899)	130 (4.3%)	629 (7.8%)	759 (6.8%)	
TNM_CLIN_N	1	592 (19.7%)	1630 (20.8%)	2222 (20.5%)	<0.001
	2	1322 (44.0%)	2520 (32.2%)	3842 (35.4%)	
	3	56 (1.9%)	50 (0.6%)	106 (1.0%)	
	0	1036 (34.5%)	3634 (46.4%)	4670 (43.1%)	
TNM_CLIN_T	1	280 (9.3%)	908 (11.6%)	1188 (11.0%)	<0.001
	2	766 (25.6%)	2270 (29.1%)	3036 (28.1%)	
	3	554 (18.5%)	1129 (14.5%)	1683 (15.6%)	
	4	1301 (43.4%)	3096 (39.6%)	4397 (40.7%)	
	X	93 (3.1%)	400 (5.1%)	493 (4.6%)	
	0	3 (0.1%)	6 (0.1%)	9 (0.1%)	
TNM_PATH_STAGE_GROUP	3	458 (15.0%)	1957 (24.3%)	2415 (21.7%)	<0.001
	4	1228 (40.3%)	5776 (71.6%)	7004 (63.0%)	
	Other(IS8899)	1360 (44.6%)	330 (4.1%)	1690 (15.2%)	
TNM_PATH_M	1	4 (100.0%)	11 (100.0%)	15 (100.0%)	
TNM_PATH_N	1	443 (17.2%)	1962 (24.7%)	2405 (22.9%)	<0.001
	2	791 (30.8%)	3591 (45.3%)	4382 (41.7%)	
	3	14 (0.5%)	64 (0.8%)	78 (0.7%)	
	X	901 (35.0%)	249 (3.1%)	1150 (10.9%)	
	0	423 (16.4%)	2066 (26.0%)	2489 (23.7%)	
TNM_PATH_T	1	273 (10.6%)	1073 (13.4%)	1346 (12.7%)	<0.001
	2	422 (16.3%)	1817 (22.8%)	2239 (21.2%)	
	3	286 (11.1%)	1332 (16.7%)	1618 (15.3%)	

	4	720 (27.8%)	3687 (46.2%)	4407 (41.7%)	
	X	881 (34.1%)	60 (0.8%)	941 (8.9%)	
	0	4 (0.2%)	16 (0.2%)	20 (0.2%)	
TNM_EDITION_NUMBER	7	3048 (99.9%)	8068 (100.0%)	11,116 (100.0%)	0.103
	*	3 (0.1%)	2 (0.0%)	5 (0.0%)	
LYMPH_VASCULAR_INVASION	Not present	1272 (41.7%)	4964 (61.5%)	6236 (56.1%)	<0.001
	Present	457 (15.0%)	2166 (26.8%)	2623 (23.6%)	
	Not applicable	3 (0.1%)	3 (0.0%)	6 (0.1%)	
	Unknown	1319 (43.2%)	937 (11.6%)	2256 (20.3%)	
REGIONAL_NODES_EXAMINED	Mean/StdErr	18.8/0.4	33.3/0.2	29.4/0.2	<0.001
	Median/Min/Max	7.0/0.0/90.0	30.0/0.0/90.0	27.0/0.0/90.0	
RNE (cat.)	No nodes examined	381 (12.5%)	1971 (24.4%)	2352 (21.1%)	<0.001
	Nodes examined	1280 (42.0%)	5656 (70.1%)	6936 (62.4%)	
	Unknown	1390 (45.6%)	443 (5.5%)	1833 (16.5%)	
REGIONAL_NODES_POSITIVE	Mean/StdErr	2.3/0.1	2.4/0.0	2.4/0.0	0.850
	Median/Min/Max	1.0/0.0/57.0	1.0/0.0/90.0	1.0/0.0/90.0	
RNP (cat.)	No nodes positive	1368 (44.8%)	433 (5.4%)	1801 (16.2%)	<0.001
	Nodes positive	1653 (54.2%)	7620 (94.4%)	9273 (83.4%)	
	Unknown	30 (1.0%)	17 (0.2%)	47 (0.4%)	
CS_METS_AT_DX	No	3051 (100.0%)	8070 (100.0%)	11,121 (100.0%)	
CS_METS_DX_BONE	None	3051 (100.0%)	8070 (100.0%)	11,121 (100.0%)	
CS_METS_DX_BRAIN	None	3051 (100.0%)	8070 (100.0%)	11,121 (100.0%)	
CS_METS_DX_LIVER	None	3051 (100.0%)	8070 (100.0%)	11,121 (100.0%)	
CS_METS_DX_LUNG	None	3051 (100.0%)	8070 (100.0%)	11,121 (100.0%)	
REASON_FOR_NO_SURGERY	Surgery of the primary site wa	1671 (54.8%)	8070 (100.0%)	9741 (87.6%)	<0.001
	Surgery not performed because	1161 (38.1%)		1161 (10.4%)	
	Surgery was not recommended/pe	91 (3.0%)		91 (0.8%)	
	Surgery not performed because	2 (0.1%)		2 (0.0%)	
	Surgery was recommeded by phys	12 (0.4%)		12 (0.1%)	
	Surgery was recommended but wa	93 (3.0%)		93 (0.8%)	
	Surgery was recommended but u	6 (0.2%)		6 (0.1%)	
	Unknown if surgery was recomme	15 (0.5%)		15 (0.1%)	
Urban	Rural	150 (5.4%)	643 (8.0%)	793 (7.3%)	<0.001
	Urban	2653 (94.6%)	7427 (92.0%)	10,080 (92.7%)	
DX_RX_STARTED_DAYS	Mean/StdErr	43.4/0.7	39.9/0.4	40.8/0.3	<0.001
	Median/Min/Max	37.0/0.0/1124.0	35.0/0.0/812.0	36.0/0.0/1124.0	
RX_SUMM_SURGICAL_ MARGINS	No residual tumor All margins	1371 (44.9%)	6798 (84.2%)	8169 (73.5%)	<0.001
	Residual tumor NOS Involveme	137 (4.5%)	538 (6.7%)	675 (6.1%)	
	Microscopic residual tumor Can	155 (5.1%)	695 (8.6%)	850 (7.6%)	
	Macroscopic residual tumor Gr	8 (0.3%)	39 (0.5%)	47 (0.4%)	
	No primary site surgery	1380 (45.2%)		1380 (12.4%)	
RX_SUMM_SURGICAL_ MARGINS_Recoded	negative	1371 (82.0%)	6798 (84.2%)	8169 (83.9%)	0.027

	positive	300 (18.0%)	1272 (15.8%)	1572 (16.1%)	
RX_SUMM_RADIATION	None	401 (13.1%)	2386 (29.6%)	2787 (25.1%)	<0.001
	Beam radiation	2626 (86.1%)	5612 (69.5%)	8238 (74.1%)	
	Radiation therapy NOS	24 (0.8%)	72 (0.9%)	96 (0.9%)	
Induction_chemo	No	1442 (80.3%)	2738 (92.2%)	4180 (87.7%)	<0.001
	Yes	354 (19.7%)	231 (7.8%)	585 (12.3%)	
