# Supplementary material for: Retrospective Cohort Study on the Impact of Travel Distance on Late-Stage Oral Cancer Treatment and Outcomes: An NCDB Analysis
Source: Cancers (Basel). 2024 Aug 2;16(15):2750. doi: 10.3390/cancers16152750 (PMC11311623; doi:10.3390/cancers16152750)
Supplement: Supplementary file 1 [file cancers-16-02750-s001.zip › Harris et al Supplemental Table S2 080124.pdf]

**Harris et al Supplemental Table S2. Multivariable Cox Hazard Ratio Modeling Adjustment Set.**

| Variable |
|---|
| travel distance |
| sex |
| age |
| race |
| treatment |
| facility type |
| urban/rural |
| insurance type |
| regional node status |
